# Supplementary material for: Socioeconomic inequality in knowledge about HIV/AIDS over time in Ethiopia: A population-based study
Source: PLOS Glob Public Health. 2023 Oct 31;3(10):e0002484. doi: 10.1371/journal.pgph.0002484 (PMC10617701; doi:10.1371/journal.pgph.0002484)
Supplement: S1 Checklist — (DOCX) [file pgph.0002484.s001.docx]

STROBE Statement—checklist of items that should be included in reports of observational studies

|  | Item No. | Recommendation | Page  No. | Relevant text from manuscript |
| --- | --- | --- | --- | --- |
| **Title and abstract** | 1 | (*a*) Indicate the study’s design with a commonly used term in the title or the abstract | 1 & 2 | A population-based and cross-sectional study |
|  |  | (*b*) Provide in the abstract an informative and balanced summary of what was done and what was found | 2 | Socioeconomic inequality in comprehensive knowledge about HIV/AIDS was quantified. The Erreyger’s concentration index was 0.251, 0.239, and 0.201 in 2005, 2011, and 2016, respectively. |
| Introduction | | | |  |
| Background/rationale | 2 | Explain the scientific background and rationale for the investigation being reported | 3 | Evidence has been argued that assessing the extent and sources of socioeconomic inequality discloses the hidden disparity in knowledge about HIV/AIDS. Investigating inequality in knowledge about HIV/AIDS has several benefits, including: first, it helps to develop and provide tailored interventions by reaching individuals with low service coverage. Second, minimise health care costs by reducing the number of people who take prophylaxis and subsequent ART by preventing people from exposing themselves to risky sexual behaviour. Third, promote social justice in the community by highlighting the distribution of resources and opportunities among different groups. Fourth, show policies and practises that perpetuate disparities based on social determinants of health that can inform policymakers and programme officers about the need for targeted services towards universal health coverage (UHC). |
| Objectives | 3 | State specific objectives, including any prespecified hypotheses | 4 | First, the study quantified the level and change of disparity across time in each social stratum. Second, the study assessed socioeconomic inequality and contributors using the concentration index and decomposition approach over time. |
| Methods | | | |  |
| Study design | 4 | Present key elements of study design early in the paper | 4 | We approached cross-sectional design using population-based data from the Ethiopian Demographic and Health Surveys (EDHS) from 2005 to 2016. |
| Setting | 5 | Describe the setting, locations, and relevant dates, including periods of recruitment, exposure, follow-up, and data collection | 4 | The study represents Ethiopian population. Ethiopia is one of east African countries. The sample size was 19541 in 2005, 29383 in 2011, and 27261 in 2016. Enumeration areas were listed from November 2004 to January 2005, September 2010 to January 2011, and September to December 2015 for the 2005, 2011, and 2016 surveys, respectively. An enumeration area is a primary sampling unit from which households and study participants were recruited. Then, the data collection period from study participants was conducted from April to August 2005, December 2010 to June 2011, and January to June 2016, respectively, for the 2005, 2011, and 2016 reports. The data collection period is the time when study participants were recruited. |
| Participants | 6 | (*a*) *Cohort study*—Give the eligibility criteria, and the sources and methods of selection of participants. Describe methods of follow-up  *Case-control study*—Give the eligibility criteria, and the sources and methods of case ascertainment and control selection. Give the rationale for the choice of cases and controls  *Cross-sectional study*—Give the eligibility criteria, and the sources and methods of selection of participants | 4 | Data sources were population-based data from the Ethiopian Demographic and Health Surveys (EDHS) from 2005 to 2016. Adults aged 15 to 49 years were the study population. Participants were selected using multistage sampling technique. EDHS conducted a two-stage cluster sampling cross-sectional study, in which samples are stratified, clustered, and selected in two stages. The base for stratification was urban and rural, which are clustered in nine regions and two city administrations. |
|  |  | (*b*) *Cohort study*—For matched studies, give matching criteria and number of exposed and unexposed  *Case-control study*—For matched studies, give matching criteria and the number of controls per case |  | N/A |
| Variables | 7 | Clearly define all outcomes, exposures, predictors, potential confounders, and effect modifiers. Give diagnostic criteria, if applicable | 5 | Comprehensive knowledge of HIV/AIDS was the outcome variable. Socioeconomic and demographic variables were independent variables |
| Data sources/ measurement | 8* | For each variable of interest, give sources of data and details of methods of assessment (measurement). Describe comparability of assessment methods if there is more than one group | *5* | A series of questions were used to generate the level of knowledge. All adults (men and women) who had ever heard of HIV/AIDS were asked five questions. These are knowing about the two most common methods to prevent HIV/AIDS infection (consistent condom use and having one uninfected sexual partner), providing the correct answer to the question, Can a healthy-looking person have HIV/AIDS? and rejecting the two misconceptions about HIV/AIDS **(**a person can get HIV from a mosquito bite and a person can get HIV by sharing a meal with people living with HIV). |
| Bias | 9 | Describe any efforts to address potential sources of bias | 6 | To assure the quality of the data, EDHS accomplished well-organized fieldwork, which involved a supervisor, a field editor, interviewers, biomarker technicians, and a driver. Training of the fieldwork team and ongoing supervision were conducted. Additionally, data quality was assured with standardized and translated tools into several local languages, data collectors with technology monitors, and appropriate software for data entry. Data editing was accomplished using Census and Survey Processing System software. All procedures contributed in minimizing risk of bias. |
|  |  |  |  |  |
| Study size | 10 | Explain how the study size was arrived at |  | 4. Figure 1 displays the flow chart of sample for the analysis. |

Continued on next page

| Quantitative variables | 11 | Explain how quantitative variables were handled in the analyses. If applicable, describe which groupings were chosen and why | 5 | Socioeconomic and demographic variables were independent variables. |
| --- | --- | --- | --- | --- |
| Statistical methods | 12 | (*a*) Describe all statistical methods, including those used to control for confounding |  | All frequency distribution and advanced analysis results were weighted estimates. A percentage was calculated for each of the measurement indicators of outcome and exploratory variables. Findings were presented in tables and figures. To see socioeconomic inequality and contributors, a concentration curve (CC), Erreygers’ concentration index (ECI), and decomposition of the ECI were performed. |
|  |  | (*b*) Describe any methods used to examine subgroups and interactions | N/A |  |
|  |  | (*c*) Explain how missing data were addressed | 6 | Missing data were managed through missing completely at random technique. |
|  |  | (*d*) *Cohort study*—If applicable, explain how loss to follow-up was addressed  *Case-control study*—If applicable, explain how matching of cases and controls was addressed  *Cross-sectional study*—If applicable, describe analytical methods taking account of sampling strategy | 7 | The multistage survey design and sampling weights were accounted for in the descriptive and analytical results because EDHS data is hierarchical in nature. |
|  |  | (*e*) Describe any sensitivity analyses | N/A |  |
| Results | | | | |
| Participants | 13* | (a) Report numbers of individuals at each stage of study—eg numbers potentially eligible, examined for eligibility, confirmed eligible, included in the study, completing follow-up, and analysed | 7 | Originally, adolescents and adults aged from 15 to 49 years old were 19541 in 2005, 29383 in 2011, and 27261 in 2016. However, after managing incomplete data in some variables, the final sample size became 18,818 for 2005, 29264 for 2011 and no incomplete response in 2016 (no change in sample size). |
|  |  | (b) Give reasons for non-participation at each stage |  |  |
|  |  | (c) Consider use of a flow diagram | 4 | Figure 1 |
| Descriptive data | 14* | (a) Give characteristics of study participants (eg demographic, clinical, social) and information on exposures and potential confounders | 8 | Table 1 |
|  |  | (b) Indicate number of participants with missing data for each variable of interest | 4 | Figure 1 |
|  |  | (c) *Cohort study*—Summarise follow-up time (eg, average and total amount) | N/A |  |
| Outcome data | 15* | *Cohort study*—Report numbers of outcome events or summary measures over time | *N/A* |  |
|  |  | *Case-control study—*Report numbers in each exposure category, or summary measures of exposure | *N/A* |  |
|  |  | *Cross-sectional study—*Report numbers of outcome events or summary measures | *8* | *Table 1* |
| Main results | 16 | (*a*) Give unadjusted estimates and, if applicable, confounder-adjusted estimates and their precision (eg, 95% confidence interval). Make clear which confounders were adjusted for and why they were included |  |  |
|  |  | (*b*) Report category boundaries when continuous variables were categorized | 8 | In Table 1, age category |
|  |  | (*c*) If relevant, consider translating estimates of relative risk into absolute risk for a meaningful time period | N/A |  |

Continued on next page

| Other analyses | 17 | Report other analyses done—eg analyses of subgroups and interactions, and sensitivity analyses or other analysis | 9-17 | Socioeconomic inequality and decomposition analysis |
| --- | --- | --- | --- | --- |
| Discussion | | | | |
| Key results | 18 | Summarise key results with reference to study objectives | 18 | Significant socioeconomic inequality was observed in comprehensive knowledge about HIV/AIDS that gradually declined between 2005 and 2016, |
| Limitations | 19 | Discuss limitations of the study, taking into account sources of potential bias or imprecision. Discuss both direction and magnitude of any potential bias | 19 | The parameters of comprehensive knowledge of HIV/AIDS may be prone to recall bias because they were based on respondents’ self-report. |
| Interpretation | 20 | Give a cautious overall interpretation of results considering objectives, limitations, multiplicity of analyses, results from similar studies, and other relevant evidence | 19 | Socioeconomic-related inequality in comprehensive knowledge is woven deeply over time in Ethiopia though this disparity has been decreased minimally. |
| Generalisability | 21 | Discuss the generalisability (external validity) of the study results | 19 | The current findings truly represent the target population because the response rate was non-significant. |
| Other information | |  | | |
| Funding | 22 | Give the source of funding and the role of the funders for the present study and, if applicable, for the original study on which the present article is based | 20 | Authors have no received fund to conduct this specific review |

*Give information separately for cases and controls in case-control studies and, if applicable, for exposed and unexposed groups in cohort and cross-sectional studies.

**Note:** An Explanation and Elaboration article discusses each checklist item and gives methodological background and published examples of transparent reporting. The STROBE checklist is best used in conjunction with this article (freely available on the Web sites of PLoS Medicine at http://www.plosmedicine.org/, Annals of Internal Medicine at http://www.annals.org/, and Epidemiology at http://www.epidem.com/). Information on the STROBE Initiative is available at www.strobe-statement.org.
