# Supplementary material for: Socioeconomic inequality in knowledge about HIV/AIDS over time in Ethiopia: A population-based study
Source: PLOS Glob Public Health. 2023 Oct 31;3(10):e0002484. doi: 10.1371/journal.pgph.0002484 (PMC10617701; doi:10.1371/journal.pgph.0002484)
Supplement: Attachment — Submitted filename: Response to reviewers.docx [file pgph.0002484.s003.docx]

**Authors’ point-by-point response**

## Manuscript submission ID: [PGPH-D-23-01054] - [EMID:692bb6feec0916df]

Socioeconomic inequality in knowledge about HIV/AIDS over time in Ethiopia: a population-based study

Dear Dr Sarah E. Brewer,

Thank you for providing us the opportunity to revise our manuscript based on helpful comments and suggestions. We appreciate the time and effort you and reviewers dedicate to providing comments and suggestions. In our revision submission, we have added supplement ideas, made extensive grammatical and stylistic revision to ensure clarity and standard English throughout the manuscript, and explained more on some points to strength the findings based on the reviewers’ comments. We have highlighted the changes in the main manuscript with track change (Revised Manuscript with Track Changes).

The following are the authors’ point-by-point responses (the academic editor’s and reviewers’ comments are **numbered,** and the authors’ responses are **underlined in *italic* format**).

Reviewer #1:

1. I congratulate the research team for working on HIV knowledge inequality across different variables. As standard concentration index inclusive of corrected indices are directly impacted by sample size and sample weights, I have noted with delight that the researchers have duly ensured that the sample weights were considered in their statistical analytic work.

*Authors’ response:* *Thank you so much for the overall feedback and appreciation.*
2. Page 4: Methodology—second sentence: revise to read: ‘’we used a cross-sectional design’’.

*Authors’ response:* *Thank you so much.* *We have revised it as per your suggestion.*
3. Page 18: Under the limitation of the study the last sentence appeared confusing: “Furthermore, the current findings truly represent the target population because response rate was non-significant”. Shouldn’t the statement be referring to ‘’ non-response rate being non-significant rather than response rate’’?

*Authors’ response:* *Thank you so much for this correction. Yes, we mean the non-response rate was non-significant. We revised this accordingly.*
4. Align the text in conclusion in the abstract with the conclusion in the main write up on page 18.

*Authors’ response:* *Thank you so much. We have revised it as per your recommendation.*

Reviewer #2:

This manuscript can be published. This is nicely written. This is a population-based survey. It has a great value. The statistical part is nicely written based on the objective of the study.

*Authors’ response:* *Thank you so much for the appreciation.*

Reviewer #3:

This is a very good study because if individual are informed about HIV/AIDS and how to prevent or what to do when infected, it is likely they engage in the behavior that will ensure they stay safe. However, knowledge doesn't significantly, alone transform to practice so the author would have tried to check if practices are also difference according to the difference in knowledge. For instance, attitudes towards the use of condoms and early diagnosis rate. Furthermore, it is expected that knowledge and preventive practices will vary across the age group so it is important to try and focus on a particular target group.

*Authors’ response:* Thank you so much for the appreciation and for raising very important issues. We agreed that the difference in knowledge may result in differences in behavioral practices and service utilization. This hypothesis by itself can be another study. As you read our manuscript, the aim of the current study was to assess socioeconomic inequality in comprehensive knowledge about HIV/AIDS over time, which is the outcome variable of the current study. The current study could not assess the impact of knowledge and attitude towards condom use because condom use was not the outcome variable of the current study. However, we agreed that these issues should be examined in the future.

Reviewer #4:

1. The topic covered by the article is very salient as it focuses on socioeconomic inequality in comprehensive knowledge on HIV/AIDS over time in Ethiopia. It adds to the body of knowledge on this area and would be a useful resource to health practitioners as they design health programs to improve knowledge on HIV/AIDS. These are several recommendations to improve the quality of the manuscript.

*Authors’ response:* *Thank you so much for the appreciation and constructive comments.*

2. The article feels rushed and incohesive, with a distinct difference in the writing styles from one section to the next (e.g., use of % then percentage points). The submission would be improved if the authors reviewed grammar, revised and reordered several awkward sentences which hinder clarity, spelt out acronyms when they are first mentioned and reviewed the writing styles in the different sections.
*Authors’ response:* *Dear reviewer, We extensively revised the English edition. To add one point on the use of ‘% and percentage point’, the purpose where we mentioned % and percentage point was different. We have used percentage point in Page 11 of the Revised Manuscript with Clean File for the interpretation purpose of the coefficient (*β).
3. The age range of the sample was 15-49 years and is representative of population in that age group. It does not represent the Ethiopians 50 years and older, so not all adults.

*Authors’ response:* *Thank you so much. We revised this as our study population is adults aged 15 to 49 years old.*
4. Was comprehensive knowledge scored 0-5 based on the number of questions answered correctly?

a. How many options were provided for each question?
b. Under results, comprehensive knowledge was given in percentages, what score equates to comprehensive knowledge?

*Authors’ response:* *Comprehensive knowledge of HIV/AIDS was the outcome variable. A series of questions were used to generate the level of knowledge among adults who had ever heard of HIV/AIDS. These questions included knowing about the two most common methods to prevent HIV/AIDS infection (‘consistent condom use’ and ‘having one uninfected sexual partner’), providing the correct answer to the question ‘Can a healthy-looking person have HIV/AIDS?’ and rejecting the two misconceptions about HIV/AIDS* ***(‘****a person can get HIV from a mosquito bite’ and ‘a person can get HIV by sharing a meal with people living with HIV’). Each question has three alternatives to be answered, including: yes (coded as 1), no (coded as 0), and do not know (also coded as 0). Those who answered yes for each question were considered to have comprehensive knowledge about HIV/AIDS (Page 5). For further clarification and supportive findings, we also added the percentage distribution of HIV/AIDS awareness, knowledge specific questions, comprehensive knowledge about HIV/AIDS in Table 1 (Page 8). Overall, we estimated proportion of adults aged 15 to 49 years having comprehensive knowledge about HIV/AIDS. We explicitly stated in the revised submission.*

5. What was the Census and Survey Processing System software used? It may not be necessary to include this statement in your manuscript but as it is included, clarification would be useful.

*Authors’ response:* *Thank you so much. We agreed that it was not necessary mentioning it in this manuscript, and we deleted it.*
6. Does this study include the limitation that “it does not provide causal evidence about whether the issues outlined are the principal drivers of the observed inequality” (Chirwa et al. 2019). Chirwa GC, Sithole L, Jamu E. Socio-economic Inequality in Comprehensive Knowledge about HIV in Malawi. Malawi Med J. 2019 Jun;31(2):104-111. doi: 10.4314/mmj.v31i2.1. PMID: 31452842; PMCID: PMC6698630.
*Authors’ response:* *Thank you so much. Yes, this evidence supports our study. We cited it in the limitation section as well.*

7. By including some background on Ethiopia, its population, level of education across its population, development indices and impact of HIV/AIDS in the population, the authors can assist readers to contextualize their research.

*Authors’ response:* *Thank you so much. We have added about the Ethiopian contexts (Page 4).*

8. Under statistical analysis, referencing other studies which used similar methodologies to analyze demographic health survey days would be useful.

*Authors’ response:* *Thank you so much. We cited some previous studies for the support of the current analysis (Page 7).*

9. The discussion can be strengthened by elaborating on and comparing the findings to other published studies in Ethiopia and other countries in the region and expanding on the recent public health strategies being used in Ethiopia.

*Authors’ response:* *Thank you so much. The discussion section is revised based on your suggestions.*

Reviewer #5: Summary:
1. The authors have made an excellent effort to find out the demographic and socio-economic factors contributing to the inequality in the knowledge of HIV/AIDS among Ethiopian population. The data for the study was obtained from 2005, 2011, and 2016 population-based health survey. The study concluded that the group with higher incomes had a greater concentration of comprehensive HIV/AIDS knowledge and socioeconomic disparities in comprehensive knowledge of HIV/AIDS have decreased over time.

*Authors’ response:* *Thank you so much for the appreciation.*
2. Though the authors have done extensive analysis of the available data, since the authors have done secondary analysis of the already available data source (NHS), I feel it does not fall under the category of original research article.

*Authors’ response:* Dear reviewer, the data sources for our study are demographic health survey data sets. Demographic health survey data is collected from the population through the survey method. The Demohraphic Health Survey comprises more than 400 health problems across 90 countries ([*https://dhsprogram.com/*](https://dhsprogram.com/)). Many other original studies have also been conducted using the Demographic Health Survey. We believe the category of the current study is original research articles because other categories, such as reviews, are not appropriate for the current kind of studies.

3. Kindly clarify Objectives: Needs to be refined further

*Authors’ response:* *Thank you so much. We have refined it.*
4. Study design: Since the data is taken at different time points and compared, can the authors still consider it as a cross-sectional study?

*Authors’ response:* Dear reviewer, data was not collected sequentially from the same respondents. The study participants for 2005, 2011, and 2016 data were different individuals. The current study estimates the proportion of individuals with comprehensive knowledge across different socioeconomic categories in 2005, 2011, and 2016. A prevalence or proportion study is usually estimated from a cross-sectional study, in which data from individuals is collected at a point in time (no follow-up). Many other studies conducted using Demographic Health Survey data set are followed similar approach and agreed that the design is cross-sectional.
5. Results: Tables 1, 2 and 3 are too long with too much information in the same table, making it difficult for the readers to comprehend.

*Authors’ response:* We understand that these tables contain a lot of information because they represent 2005, 2011, and 2016. If we prepare separated tables for 2005, 2011, and 2016, the number of tables will be nine. Thus, we prefer to present related findings in one table for all years, which allows us to see differences and similarities in one table.
