# Supplementary material for: Socioeconomic inequality in knowledge about HIV/AIDS over time in Ethiopia: A population-based study
Source: PLOS Glob Public Health. 2023 Oct 31;3(10):e0002484. doi: 10.1371/journal.pgph.0002484 (PMC10617701; doi:10.1371/journal.pgph.0002484)
Supplement: Attachment — Submitted filename: RESPON~1.DOC [file pgph.0002484.s004.DOC]

**PGPH-D-23-01054R1
Socioeconomic inequality in knowledge about HIV/AIDS over time in Ethiopia: a population-based study**,

**Authors’ point-by-point response**

We would like to extend our gratitude to the editorial board and the expert reviewers for their time in reviewing our manuscript and providing us with the opportunity to revise it. We believe the reviewers provided invaluable feedback and which has helped to improve the manuscript. We hope you now find it suitable for publication. In our revision submission, we have considered all comments and suggestions. We have submitted the changes with track changes in the ‘Revised Manuscript with Track Changes'. We have also submitted a clean file ‘Manuscript’.

The following are the authors’ point-by-point responses (the editor’s and reviewers’ comments are **numbered,** and the authors’ responses are **bold and underlined in *italic* format**).

EDITOR comments:

1. Thank you for your thorough revisions of the manuscript.  Reviewer #4 has highlighted a few revisions for consistency (e.g., DHS vs. EDHS) and strength of the discussion that warrant a few more minor revisions.  I hope the authors will choose to make these revisions.  If I am available, I would agree to handle the revised manuscript.

***Authors’ response: Thank you so much for this suggestion. We have made the necessary revision. Regarding DHS vs. EDHS, we have revised except in the ‘Ethical Considerations’ section because Ethical approval was obtained from DHS. DHS program has collected, analysed, and disseminated data on population, health, HIV, and nutrition in over 90 countries, including Ethiopia. Data related to EDHS related data are available on the DHS website, and the ethical approval was obtained from DHS.***

2. Please also note that there is disagreement about your data accessibility. Please clearly state how data from this study is accessible or justification for not making data publicly available in your response letter.

***Authors’ response: We utilized data from the Demographic Health Survey (DHS) Program, previously described. The Ethiopian demographic health surveys data we obtained from DHS was acquired through a formal request from DHS. The website for the DHS Program is*** [***https://dhsprogram.com/***](https://dhsprogram.com/)***.***

3. Journal Requirements:

   Please review your reference list to ensure that it is complete and correct. If you have cited papers that have been retracted, please include the rationale for doing so in the manuscript text, or remove these references and replace them with relevant current references. Any changes to the reference list should be mentioned in the rebuttal letter that accompanies your revised manuscript. If you need to cite a retracted article, indicate the article’s retracted status in the References list and also include a citation and full reference for the retraction notice.

***Authors’ response: Thank you for the reminder. We have reviewed and addressed minor issues, such as adding url, date, correcting punctuation, in the reference lists. Furthermore, we have verified that no retracted articles are included in the reference lists, as confirmed during our recheck of all references on 19 September 2023.***

**Review Comments to the Author**

**Reviewer #1**:

1. Congratulations

***Authors’ response: Thank you so much for reviewing our manuscript and appreciating our works.***

**Reviewer #4:**

1. The manuscript flow has been much improved since your first submission. Congratulations on your hard work to revise the piece and for incorporating the comments of the reviewers.

***Author’s response: Thank you so much for reviewing our manuscript and the appreciation.***

2. Minor Revisions Recommended
   There are several instances of awkward sentences and incorrect grammar to be fixed.
   • For example – “by preventing individuals from engaging in risky sexual behavior”.

***Authors’ response: Thank you so much for comments. We read the manuscript repeatedly, and we have now improved well.***

3. Please be consistent with the use of EDHS or DHS.

***Authors’ response: Thank you so much. We have now revised in most part. However, there is a condition that needs to use DHS than EDHS, specifically in ‘Ethical Considerations’ sections because data related to EDHS related data are available on the DHS website, and the ethical approval was obtained from DHS.***

4. Cite the source of the information on the EDHS methodology.

***Authors’ response: Thank you so much. We have now put the citation properly.***

5. Insert a reference for the missing completely at random technique.

***Authors’ response: We have now included citation for this.***

6. Was the method used in the analysis used by other researchers analyzing DHS data? If so, give several examples.

***Authors’ response: Yes, other researchers have also used DHS data using the method used in the analysis of our study. We have already included in the ‘Statistical analysis’ section last sentence.***

7. The discussion could be strengthened by elaborating on the findings of some of the studies briefly mentioned.

• Studies revealed similar findings (17, 28).

• Other studies which found that men had better education and employment opportunities, and internet access compared to women (references 57, 58, 59).
• The outcome of the individual level approach (reference 64).

***Authors’ response: Thank you so much. We have now clearly elaborated all these.***

8. Please clarify the statement in the limitation that this study does not exactly show causation.

***Authors’ response: Thank you so much. We have now revised based on the suggestion.***

**Reviewer #5: (No Response)**

***Authors’ response: Thank you so much for reviewing our manuscript***
